# p66Shc Mediates Mitochondrial Dysfunction Dependent on PKC Activation in Airway Epithelial Cells Induced by Cigarette Smoke

**DOI:** 10.1155/2018/5837123

**Published:** 2018-04-11

**Authors:** Ming Zhang, Jingjing Tang, Hu Shan, Qiuhong Zhang, Xia Yang, Jie Zhang, Yali Li

**Affiliations:** Department of Respiratory and Critical Care Medicine, The Second Affiliated Hospital of Xi'an Jiaotong University, Xi'an, Shaanxi, China

## Abstract

Airway epithelial mitochondrial injury plays a critical role in the pathogenesis of chronic obstructive pulmonary disease (COPD). The p66Shc adaptor protein is a newly recognized mediator of mitochondrial dysfunction. However, little is known about the effect of p66Shc on airway epithelial damage in the development of COPD. The aim of the present study is to investigate the roles of p66Shc and its upstream regulators in the mitochondrial injury of airway epithelial cells (Beas-2b) induced by cigarette smoke extract (CSE). Our present study revealed that CSE increased p66Shc expression and its mitochondrial translocation in concentration and time-dependent manners in airway epithelial cells. And p66Shc siRNA significantly attenuated mitochondrial dysfunction and cell injury when airway epithelial cells were stimulated with 7.5% CSE. The total and phosphorylated expression of PKC*β* and PKC*δ* was significantly increased associated with mitochondrial dysfunction and cell injury when airway epithelial cells were exposed to 7.5% CSE. The pretreatments with pharmacological inhibitors of PKC*β* and PKC*δ* could notably suppress p66Shc phosphorylation and its mitochondrial translocation and protect the mitochondria and cells against oxidative damage when airway epithelial cells were incubated with 7.5% CSE. These data suggest that a novel PKC*β*/*δ*-p66Shc signaling pathway may be involved in the pathogenesis of COPD and other oxidative stress-associated pulmonary diseases and provide a potential therapeutic target for these diseases.

## 1. Introduction

Cigarette smoke is the major risk factor for the development of chronic obstructive pulmonary disease (COPD), which is characterized by persistent airflow limitation and pulmonary function decline [1]. Inhaled cigarette smoke primarily encounters airway epithelium and results in airway inflammation and oxidative stress [2]. Although cigarette smoke contains various chemicals including reactive oxygen species (ROS), the gaseous-phase ROS in cigarette smoke can hardly enter airway epithelial cells and certainly not the circulation [3]. Nevertheless, some studies have demonstrated that cigarette smoke upregulates ROS levels in airway epithelial cells [4, 5] and systemic levels of oxidative stress are also dramatically increased in smokers and COPD patients [6, 7]. Therefore, cigarette smoke-induced endogenous ROS in airway epithelial cells may play an important role in the pathogenesis of COPD.

The mitochondrion is an important source of endogenous ROS which plays crucial roles in a variety of physiological and pathological circumstances [8]. The mitochondria protect themselves from oxidative damage by producing antioxidative scavengers, regulating the oxidative phosphorylation process responsible for ATP generation, and exchanging mitochondrial DNA through fusion and fission events [9]. Excessive oxidative stress and/or imbalance between oxidation and antioxidation can induce mitochondrial oxidative injury. It has been reported that cigarette smoke can lead to airway epithelial mitochondrial oxidative damage [10], but the underlying mechanism is still not fully understood.

Many signaling molecules are involved in mitochondrial ROS production including adaptor protein p66Shc, protein kinase C (PKC), and cytochrome c [11–13]. p66Shc is the longest form of the adaptor protein of the ShcA family and expressed in most of mammalian tissues [11]. It has been demonstrated that p66Shc fulfills its biological function in the mitochondria, where p66Shc oxidizes cytochrome c and forms ROS by utilizing electrons from the respiratory chain [14]. During this process, the translocation of p66Shc from the cytosol to the mitochondrial intermembrane space requires its Ser36 residue phosphorylation [15]. Although p66Shc-mediated mitochondrial oxidative stress is involved in several diseases [16–20], the upstream regulation of p66Shc phosphorylation remains poorly understood. It has been reported that cJun N-terminal kinases (JNKs) regulate p66Shc Ser36 residue phosphorylation and mitochondrial ROS production under oxidative stress [21]. Some other studies have also demonstrated that proapoptotic signals activate protein kinase C (PKC) isozymes, including PKC*β* and PKC*δ*, which in turn phosphorylate p66Shc at Ser36 residue in renal tubular epithelial cells [22, 23]. However, it is still unclear whether the PKC*β*/*δ*-p66Shc signaling pathway can modulate mitochondrial dysfunction in airway epithelial cells exposed to cigarette smoke.

In this present study, we explored the effect of cigarette smoke extract (CSE) on the expression of total and phosphorylated p66Shc in airway epithelial cells and further investigated the regulatory effect of PKC*β*/*δ* on p66Shc activation and mitochondrial dysfunction during airway epithelial cell injury induced by CSE.

## 2. Methods

### 2.1. Preparation of CSE

Septwolves cigarettes (tobacco type of tar: 10 mg, nicotine content: 0.8 mg, and carbon monoxide fumes: 12 mg) were purchased from China Tobacco Fujian Industry Limited Liability Company and used to prepare CSE through a puffing mechanism mimicking a standardized human smoking pattern (volume 30 ml/puff, duration 2 s/puff, and frequency 1 puff/min) as described elsewhere with minor modifications [24]. A total of 10 puffs (300 ml) of cigarette smoke were bubbled through 10 ml RPMI 1640 medium in a glass bottle and mixed by shaking, followed by adjusting pH to 7.4. The CSE solution was passed through a 0.22 *μ*m filter to remove large particles, and this solution was defined as 100% CSE. Working concentration was made by diluting the 100% CSE with culture medium.

### 2.2. Cell Culture and Treatments

Human bronchial epithelial cells of Beas-2b were obtained from the Shanghai Cell Bank of Chinese Academy of Sciences and cultured in RPMI 1640 medium supplemented with 10% fetal bovine serum (Gibco) and 100 units/ml of penicillin/streptomycin in a humidified incubator under 5% CO_2_ at 37°C. Cells were stimulated with CSE under various concentrations and time points. In addition, cells were also stimulated with 7.5% CSE after the knockdown of p66Shc or 30 min pretreatments with PKC*β* and PKC*δ* inhibitors (Sigma). The final concentration is 10 *μ*M for the PKC*β* inhibitor (LY333531) and 5 *μ*M for the PKC*δ* inhibitor (rottlerin).

#### 2.2.1. Small Interfering RNA (siRNA) for p66Shc

Control scrambled siRNA and p66Shc siRNA (sense 5′-UGAGUCUCUGUCAUCGCUGTT-3′ and antisense 5′-CAGCGAUGACAGAGACUCATT-3′) were purchased from GenePharma Co. Ltd. (Shanghai, China) and transfected with Lipofectamine 2000 (Invitrogen) according to the manufacturer's protocol. The efficiency of knockdown was confirmed by real-time PCR and Western blot.

### 2.3. Cell Viability Assay

After the indicated treatments, cell viability was determined by the conventional 3-(4,5-dimethylthiazol-2-yl)-2,5-diphenyltetrazolium bromide (MTT) assay and expressed as a percentage of the control group. Moreover, the cells were stained with trypan blue dye at 37°C for 3 min, and viable (unstained) and nonviable (blue-stained) cells were counted to calculate their viability.

### 2.4. Apoptosis Assay

The cell surface exposure of phosphatidylserine and plasma membrane disruption was evaluated by staining with annexin V-APC and 7-AAD according to the manufacturer's protocol (KeyGEN BioTECH, China). The cells were analyzed by a FACSCalibur flow cytometer (Becton Dickinson GmbH, Heidelberg, Germany). The apoptosis rate was expressed as the percentage of annexin V-APC-positive cells to the total cells.

### 2.5. Mitochondrial Membrane Potential (MMP) Determination

The levels of MMP were determined using the FACSCalibur flow cytometer (Becton Dickinson GmbH, Heidelberg, Germany) according to the instruction of the JC-1 assay kit (Beyotime, China). JC-1 forms J-aggregates emitting red fluorescence at 590 nm in healthy mitochondria and J-monomers emitting green fluorescence at 490 nm in depolarized mitochondria, and the results were expressed as the relative ratio of red to green fluorescence intensity.

### 2.6. Measurement of Mitochondrial ROS

MitoSOX Red (Invitrogen), a live-cell permeant dye that rapidly and selectively targets the mitochondria, was used to measure the levels of mitochondrial ROS. Fluorescence intensity was detected by a laser scanning confocal microscope (Nikon C2, Japan) at 510 nm excitation and 580 nm emission wavelengths and further quantified using the Image-Pro Plus 6.0 software.

### 2.7. Intracellular ATP Detection

Intracellular ATP levels were detected using a firefly luciferase-based ATP assay kit (Beyotime, China) according to the manufacturer's instruction. Luminance was measured with a fluorescence microplate reader (Tecan Infinite M200, Switzerland). And the results of ATP were expressed relative to the control group.

### 2.8. Measurement of Cytokines

The levels of IL-6 and TNF-*α* in cell-free culture supernatants were measured using the corresponding ELISA kits (Yuanye Biological Technology Co. Ltd., Shanghai, China) according to the manufacturer's instructions.

### 2.9. Real-Time PCR

Total mRNA was extracted using the TRIzol reagent (Invitrogen), and reverse transcription was carried out using an RT-PCR kit (TaKaRa), and then p66Shc mRNA expression was measured by real-time PCR (TaKaRa). The primers were as follows: 5′-AAGTACAATCCACTCCGGAATGA-3′ (forward) and 5′-GGGCCCCAGGGATGAAG-3′ (reverse) for p66Shc and 5′- AGCGAGCATCCCCCAAAGTT-3′ (forward) and 5′-GGGCACGAAGGCTCATCATT-3′ (reverse) for *β*-actin. Results were expressed as fold differences relative to the level of *β*-actin using the 2^−△△CT^ method.

### 2.10. Western Blot

The mitochondria were isolated from airway epithelial cells by differential centrifugation as described previously [25]. The whole cells and mitochondrial fraction were homogenized in a RIPA lysis buffer containing protease inhibitors, and protein concentration was determined by a BCA protein assay kit (Beyotime, China). 40 *μ*g of protein was subjected to sodium dodecyl sulfate-polyacrylamide gel electrophoresis and transferred onto a polyvinylidene difluoride membrane. The membranes were then incubated with specific antibodies against p66Shc (1 : 1000, Abcam), p-p66Shc (S36, 1 : 1000, Abcam), PKC*β* (1 : 1000, Proteintech Group), p-PKC*β* (T642, 1 : 800, Abcam), PKC*δ* (1 : 800, Proteintech Group), p-PKC*δ* (Y311, 1 : 3000, Abcam), cytochrome c (1 : 800, Proteintech Group), GAPDH (1 : 1000, Santa Cruz), and COXIV (1 : 600, Abcam) at 4°C overnight. And the membranes were incubated with secondary antibodies for additional 2 h. Later, the bands were scanned and densitometric analysis was performed with Bandscan 5.0 software. GAPDH and COXIV were used as loading controls for the whole cellular and mitochondrial proteins, respectively, and results were expressed relative to the control.

### 2.11. Statistical Analysis

All data are presented as the mean ± standard deviation (SD) and were analyzed using SPSS 16.0 software. The analysis of data was performed using one-way analysis of variance test and LSD test. *P* < 0.05 was considered statistically significant.

## 3. Results

### 3.1. CSE Increased p66Shc Expression and Its Mitochondrial Translocation

**Figure 1:**
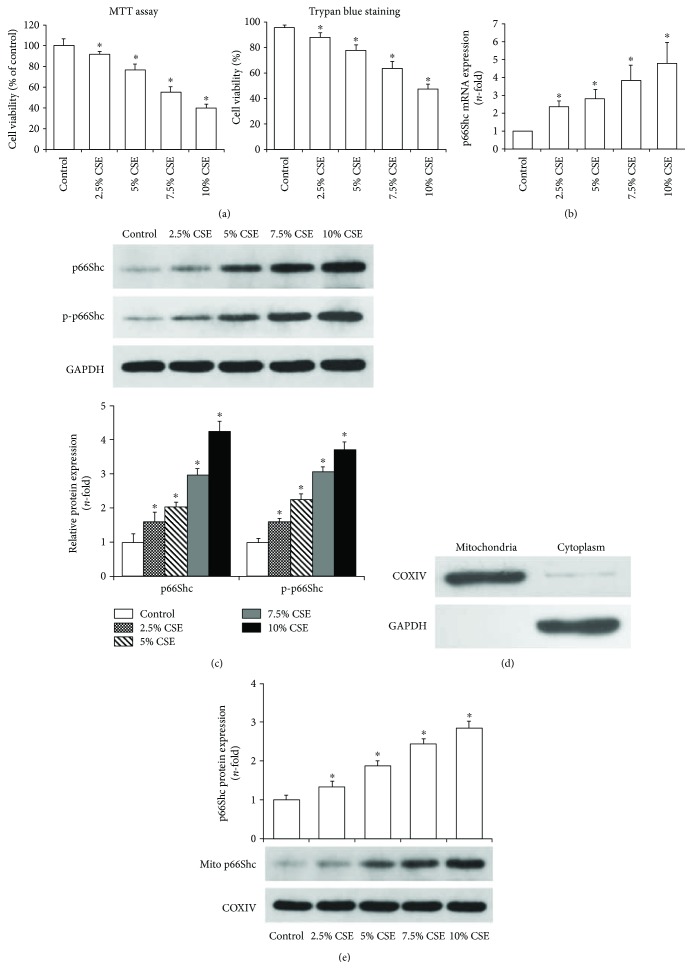
CSE increased p66Shc expression in a concentration-dependent manner in Beas-2b cells. When airway epithelial cells were exposed to CSE for 24 h, cell viability was gradually decreased at the indicated concentration (a). After 24 h stimulation with CSE (2.5–10%), real-time PCR showed that p66Shc mRNA expression was increased in a concentration-dependent manner (b). Western blot showed that the expression levels of p66Shc and p-p66Shc in the whole cell lysates were significantly increased in a concentration-dependent manner when the cells were stimulated with CSE (2.5–10%) for 24 h (c). The mitochondria were isolated from airway epithelial cells by differential centrifugation, and mitochondrial purity was confirmed by Western blot (d). p66Shc expression in the mitochondrial lysates was also significantly increased when the cells were stimulated with CSE (2.5–10%) for 24 h (e). All statistical data were obtained from three independent experiments and presented as the mean ± SD. ^∗^*P* < 0.05 versus the control group.

**Figure 2:**
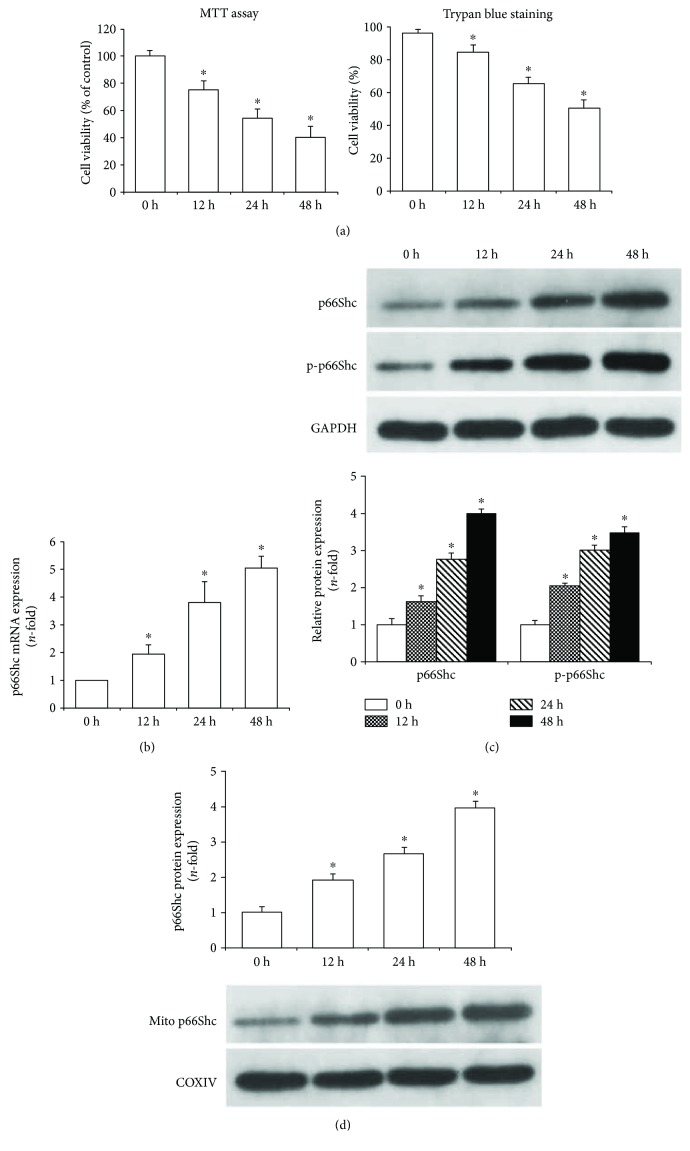
CSE increased p66Shc expression in a time-dependent manner in Beas-2b cells. Cell viabilities were determined by MTT and trypan blue exclusion assay, and they were gradually decreased when airway epithelial cells were incubated with 7.5% CSE for 0–48 h (a). After stimulation with 7.5% CSE at the indicated time points, real-time PCR showed that p66Shc mRNA expression was increased in a time-dependent manner (b). Western blot showed that the expression levels of p66Shc and p-p66Shc in the whole cell lysates (c) and p66Shc expression in the mitochondrial lysates (d) were all increased in a time-dependent manner when the cells were stimulated with 7.5% CSE at the indicated time points. All statistical data were obtained from three independent experiments and presented as the mean ± SD. ^∗^*P* < 0.05 compared to the cells treated with CSE for 0 h.

MTT and trypan blue exclusion assay showed that cell viabilities were decreased in a concentration-dependent manner when airway epithelial cells were stimulated with 2.5%–10% CSE for 24 h (*P* < 0.05, Figure 1(a)). Then the cells were stimulated with 7.5% CSE at different time points, and cell viabilities were decreased in a time-dependent manner as shown in Figure 2(a) (*P* < 0.01). Real-time PCR analysis showed that p66Shc mRNA expression was increased in concentration- and time-dependent manners when airway epithelial cells were stimulated with CSE (Figures 1(b) and 2(b), *P* < 0.05). Western blot results further revealed that the expression levels of p66Shc and p-p66Shc in the whole cell lysates were increased in concentration- and time-dependent manners when airway epithelial cells were stimulated with CSE (*P* < 0.05, Figures 1(c) and 2(c)). In addition, the mitochondria were isolated from airway epithelial cells by differential centrifugation. Mitochondrial purity was confirmed by Western blot, and the results showed that there was no cytosolic protein in the mitochondrial fraction (Figure 1(d)). And p66Shc protein expression in the mitochondrial fraction was also significantly increased in concentration- and time-dependent manners when airway epithelial cells were stimulated with CSE as shown in Figures 1(e) and 2(d) (*P* < 0.05).

### 3.2. p66Shc Silencing Attenuated Mitochondrial Dysfunction and Cell Injury Induced by CSE

**Figure 3:**
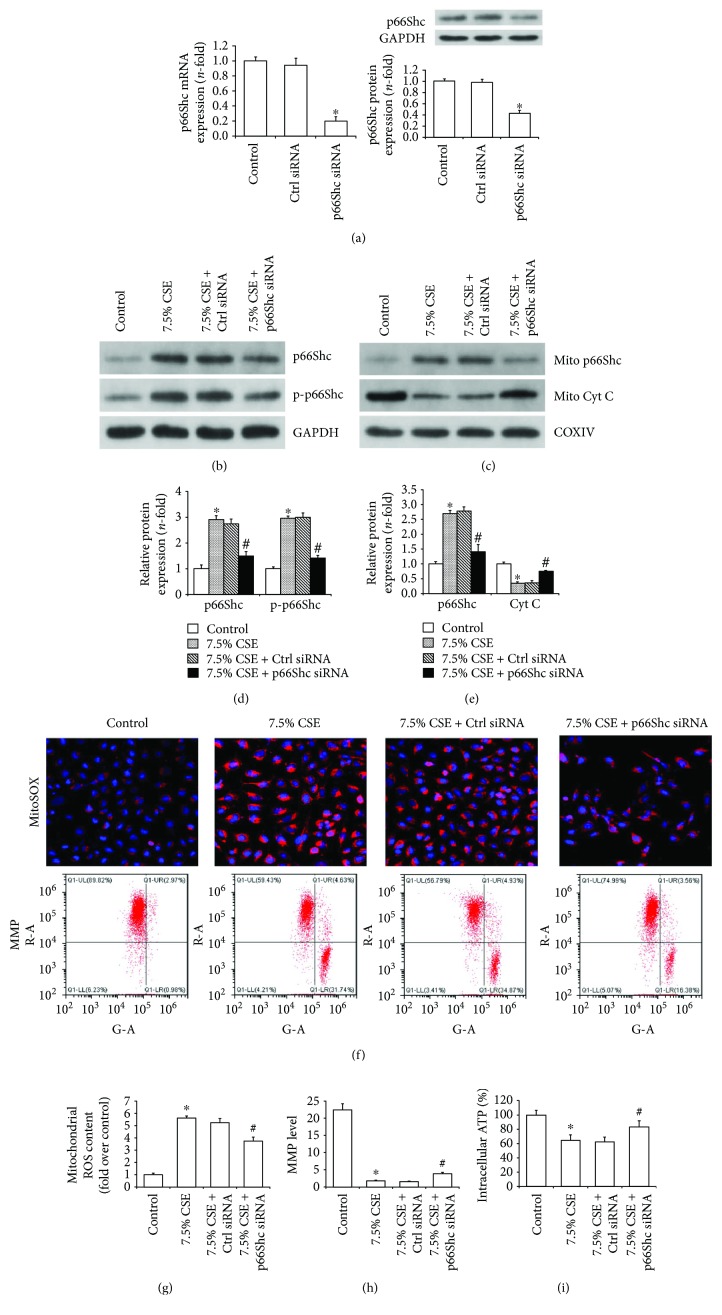
p66Shc silencing improved CSE-induced mitochondrial dysfunction of airway epithelial cells. Beas-2b cells were transiently transfected with p66Shc siRNA for 24 h, and the silencing effect was confirmed by real-time PCR and Western blot (a). Transfected Beas-2b cells were further stimulated with 7.5% CSE for 24 h, and Western blot revealed that p66Shc siRNA significantly suppressed the increasement of p66Shc and p-p6Shc expression in the whole cell lysates (b and d) and notably reduced p66Shc mitochondrial translocation and the release of cytochrome c from the mitochondria (c and e). Quantitative analysis of protein expression as shown in (d) and (e) was carried out by Bandscan 5.0 software. Mitochondrial reactive oxygen species (ROS) was observed by confocal microscopy (f: upper row), and mitochondrial membrane potential (MMP) was determined by flow cytometry (f: lower row). Quantitative analysis showed that p66Shc silencing reduced mitochondrial ROS generation (g) and elevated MMP level (h) in Beas-2b cells exposed to 7.5% CSE. Firefly luciferase-based ATP assay revealed that p66Shc siRNA increased intracellular ATP levels in Beas-2b cells stimulated with 7.5% CSE for 24 h (i). All values are showed as the mean ± SD from three independent experiments. ^∗^*P* < 0.01 versus the control group and ^#^*P* < 0.05 versus the 7.5% CSE-treated group.

In order to illustrate the role of p66Shc in airway epithelial mitochondrial injury induced by CSE, p66Shc expression was further knocked down by siRNA. Real-time PCR and Western blot displayed that the mRNA and protein expression of p66Shc were apparently downregulated by p66Shc siRNA (*P* < 0.01, Figure 3(a)). p66Shc silencing significantly reduced the increase in p66Shc and p-p66Shc protein expression in the whole cell lysates when airway epithelial cells were stimulated with 7.5% CSE (*P* < 0.01, Figures 3(b) and 3(d)). p66Shc silencing also notably suppressed p66Shc mitochondrial translocation and increased cytochrome c content in the mitochondria when airway epithelial cells were exposed to 7.5% CSE (*P* < 0.01, Figures 3(c) and 3(e)). The mitochondrial function of airway epithelial cells was assessed by a series of experiments after the intervention with p66Shc siRNA and CSE. Compared with the control values, 7.5% CSE significantly increased mitochondrial ROS content assessed by confocal microscopy and decreased MMP levels determined by flow cytometry (*P* < 0.01), and all these changes were notably attenuated in cells transfected with p66Shc siRNA (*P* < 0.05, Figures 3(f)–3(h)). ATP assay measured by a fluorescence microplate reader revealed that intracellular ATP content in cells treated with 7.5% CSE was notably decreased, and this decrease was dramatically reversed by the treatment with p66Shc siRNA (both *P* < 0.05, Figure 3(i)).

**Figure 4:**
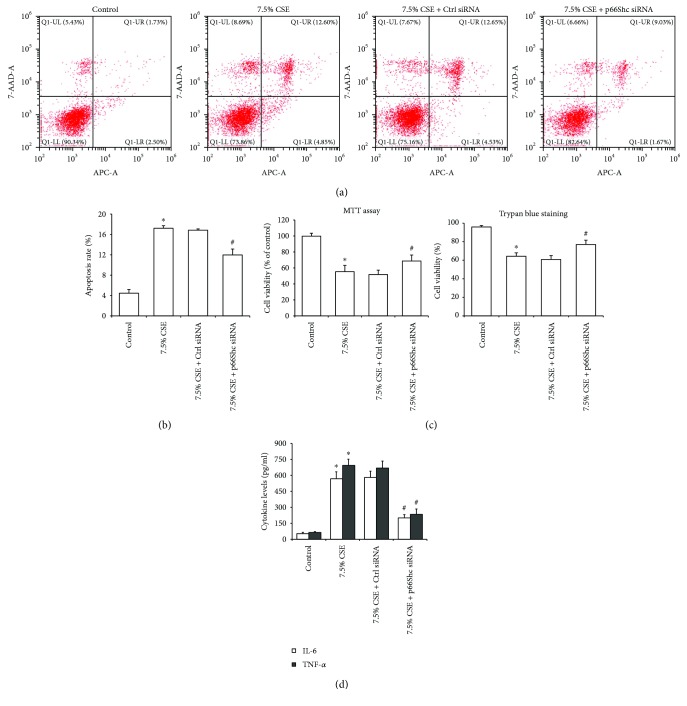
p66Shc silencing attenuated CSE-induced airway epithelial cell injury. When transfected Beas-2b cells were stimulated with 7.5% CSE for 24 h, annexin V-APC and 7-AAD-based apoptosis assay was used to determine cell apoptosis by flow cytometry (a). p66Shc silencing significantly improved cell apoptosis rate (b), cell viability (c), and culture supernatant concentration of IL-6 and TNF-*α* (d) in Beas-2b cells stimulated with 7.5% CSE. Results are expressed as the mean ± SD from three independent experiments. ^∗^*P* < 0.01 versus the control group and ^#^*P* < 0.05 versus the 7.5% CSE-treated group.

In addition, 7.5% CSE significantly increased cell apoptosis rate and decreased cell viability (*P* < 0.05), and all these changes were significantly improved by p66Shc siRNA (*P* < 0.05, Figures 4(a)–4(c)). Results of ELISA showed that 7.5% CSE notably increased the concentration of IL-6 and TNF-*α* in the cell culture supernatant, and the expression levels of these cytokines were significantly decreased following the treatment with p66Shc siRNA (*P* < 0.01, Figure 4(d)).

### 3.3. PKC*β* and PKC*δ* Modulated CSE-Induced p66Shc Activation in Airway Epithelial Cells

**Figure 5:**
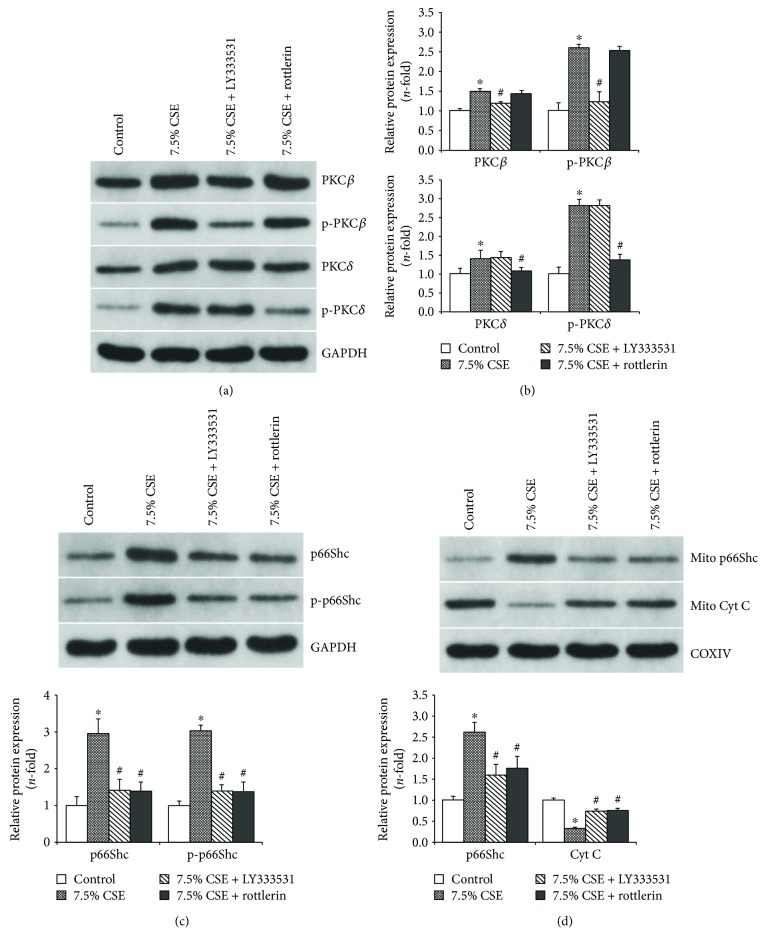
Pharmacological inhibitors of PKC*β* and PKC*δ* suppressed CSE-induced p66Shc activation and its mitochondrial translocation in airway epithelial cells. The total and phosphorylated protein expression of PKC*β* and PKC*δ* was notably increased in Beas-2b cells exposed to 7.5% CSE for 24 h, and these alterations could be downregulated by 30 min pretreatments with 10 *μ*M LY333531 and 5 *μ*M rottlerin. A representative band is shown for each condition (a), and densitometric analysis of the bands was carried out by Bandscan 5.0 software to quantify the protein expression (b). Further, Western blot analysis revealed that the pretreatments with LY333531 and rottlerin significantly decreased the protein expression of p66Shc and p-p66Shc in the whole cell lysates (c) and suppressed p66Shc mitochondrial translocation and cytochrome c release from the mitochondria (d) in Beas-2b cells incubated with 7.5% CSE for 24 h. All the data are shown as the mean ± SD from three independent experiments. ^∗^*P* < 0.05 versus the control group and ^#^*P* < 0.05 versus the 7.5% CSE-treated group.

The upstream regulatory molecules of p66Shc in airway epithelial mitochondrial injury induced by CSE have been studied by the pretreatments with PKC inhibitors. Western blot analysis showed that 7.5% CSE significantly increased the total and phosphorylated protein expression of PKC*β* and PKC*δ* (*P* < 0.05, Figures 5(a) and 5(b)). When airway epithelial cells were treated with 7.5% CSE, PKC*β* inhibitor LY333531 significantly reduced the increasement of PKC*β* and p-PKC*β* expression, and PKC*δ* inhibitor rottlerin could also suppress the increasement of PKC*δ* and p-PKC*δ* expression (*P* < 0.05, Figures 5(a) and 5(b)).

Compared with those from the CSE-treated group, the protein expression levels of p66Shc and p-p66Shc in the whole cell lysates from the LY333531 and rottlerin pretreated groups were all significantly decreased (*P* < 0.01, Figure 5(c)). Moreover, the pretreatments with LY333531 and rottlerin could significantly decrease p66Shc expression and increase cytochrome c content in the mitochondria of airway epithelial cells stimulated with 7.5% CSE (*P* < 0.01, Figure 5(d)).

### 3.4. PKC*β* and PKC*δ* Regulated CSE-Induced Mitochondrial Dysfunction and Cell Injury

**Figure 6:**
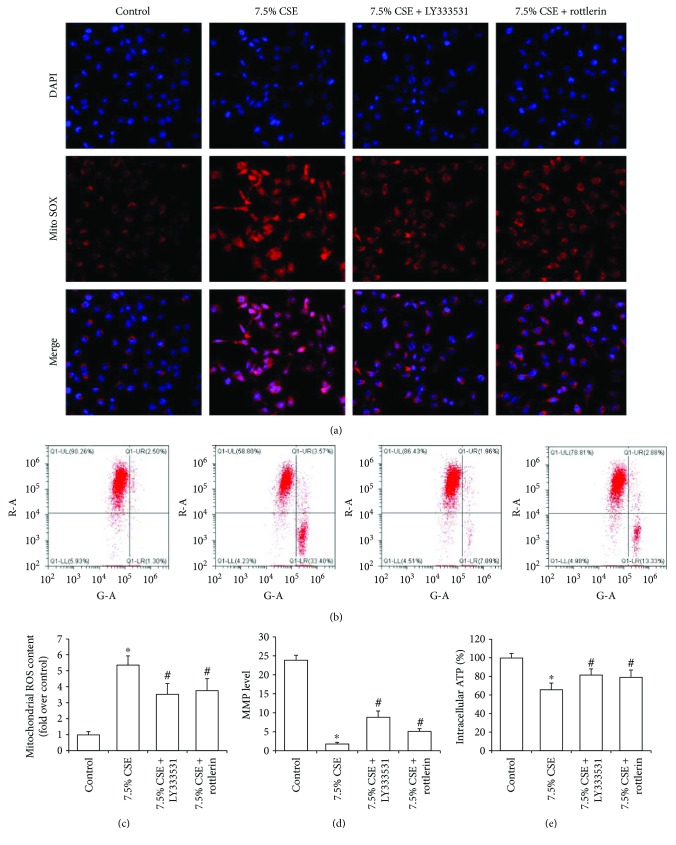
Pretreatments with PKC*β* and PKC*δ* inhibitors ameliorated CSE-induced mitochondrial damage of airway epithelial cells. Mitochondrial reactive oxygen species (ROS) was observed by confocal microscopy (a), and mitochondrial membrane potential (MMP) was determined by flow cytometry (b). Quantitative analysis showed that pretreatments with LY333531 and rottlerin reduced mitochondrial ROS content (c) and increased the levels of MMP (d) and intracellular ATP (e) in Bease-2b cells stimulated with 7.5% CSE for 24 h. All the results are shown as the mean ± SD from three independent experiments. ^∗^*P* < 0.01 versus the control group and ^#^*P* < 0.05 versus the 7.5% CSE-treated group.

Confocal microscopy and flow cytometry analysis displayed that LY333531 and rottlerin pretreatments notably decreased mitochondrial ROS content and increased MMP levels in airway epithelial cells exposed to 7.5% CSE (Figures 6(a) and 6(b)), and further quantitative analysis confirmed the protective effect of PKC*β* and PKC*δ* inhibitors (*P* < 0.05, Figures 6(c) and 6(d)). And intracellular ATP content in cells treated with 7.5% CSE and the PKC*β* inhibitor was significantly higher than that in the cells treated only with 7.5% CSE (*P* < 0.05, Figure 6(e)), and the pretreatment with the PKC*δ* inhibitor had a similar effect on intracellular ATP production (*P* < 0.05, Figure 6(e)).

**Figure 7:**
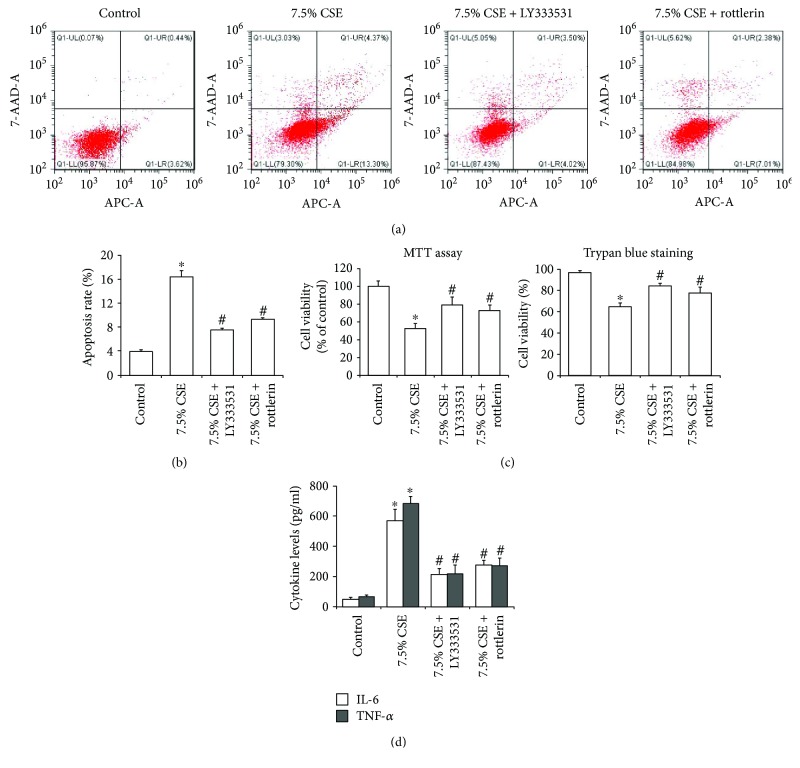
Pretreatments with PKC*β* and PKC*δ* inhibitors attenuated CSE-induced airway epithelial cell injury. Cell apoptosis was determined by flow cytometry (a), and apoptosis rates of Beas-2b cells treated with 7.5% CSE and PKC*β*/*δ* inhibitors were both significantly decreased compared to the cells treated only with 7.5% CSE (b). Pretreatments with LY333531 and rottlerin significantly upregulated cell viability (c) and downregulated culture supernatant concentration of IL-6 and TNF-*α* (d) in Beas-2b cells exposed to 7.5% CSE. Data are expressed as the mean ± SD from three independent experiments. ^∗^*P* < 0.01 versus the control group and ^#^*P* < 0.05 versus the 7.5% CSE-treated group.

Cell apoptosis and viability were further measured to illustrate the roles of the PKC*β* and PKC*δ* inhibitors in CSE-induced airway epithelial cell injury. Compared with airway epithelial cells treated only with 7.5% CSE, pretreatments with LY333531 and rottlerin could significantly decrease cell apoptosis rates and increase cell viabilities (*P* < 0.05, Figures 7(a)–7(c)). In addition, the expression levels of IL-6 and TNF-*α* in culture supernatant were significantly decreased by the pretreatments with LY333531 and rottlerin on airway epithelial cells exposed to 7.5% CSE (*P* < 0.01, Figure 7(d)).

## 4. Discussion

Our present study has demonstrated that CSE increased p66Shc expression and its mitochondrial translocation in concentration- and time-dependent manners and p66Shc siRNA attenuated mitochondrial dysfunction and airway epithelial cell injury induced by CSE. Pretreatments with pharmacological inhibitors of PKC*β* and PKC*δ* could significantly suppress p66Shc phosphorylation and its mitochondrial translocation and improve mitochondrial dysfunction when airway epithelial cells were exposed to CSE.

Cigarette smoke induces prominent oxidative stress by the production of endogenous ROS and plays an important role in the pathogenesis of COPD [1, 26]. The mitochondria are one of the main sources of endogenous ROS in eukaryotic cells [8], thus so this organelle may be an important target for cigarette smoke. As airway epithelium is the first barrier between inhaled air and the underlying lung tissues, cigarette smoke can lead to significant airway epithelial mitochondrial dysfunction [27]. However, it is still not clear how cigarette smoke induces airway epithelial mitochondrial injury.

p66Shc was identified as a negative lifespan regulator in 1999, and knocking out of this gene can prolong the lifetime of mice approximately 30% [28]. It is a newly recognized mediator of mitochondrial dysfunction, which can induce ROS formation by oxidizing cytochrome c in the mitochondria [14]. And p66Shc mediates mitochondrial dysfunction under various cellular stresses, such as ultraviolet radiation [29], angiotensin II [30], high glucose [22, 31], alcohol [32], and H_2_O_2_ [33]. Our previous study has demonstrated that p66Shc is involved in the mitochondrial injury of alveolar epithelial cells in a rat model of COPD induced by cigarette smoke exposure combined with intratracheal administration of LPS [34]. In this study, we have further found that the mRNA and protein expression of p66Shc was significantly increased in concentration- and time-dependent manners in airway epithelial cells stimulated with CSE, suggesting that p66Shc is involved in cigarette smoke-induced airway epithelial cell injury. p66Shc silencing notably decreased mitochondrial ROS production and elevated the levels of MMP and intracellular ATP in CSE-treated cells, indicating that p66Shc mediated mitochondrial dysfunction of airway epithelial cells induced by CSE. Furthermore, the degree of cell injury (cell apoptosis and viability) was consistent with p66Shc expression. In addition, it has been reported that the accumulation of mitochondrial ROS activates inflammatory pathways and promotes the production of proinflammatory cytokines including IL-6 and TNF-*α* [35]. Our present study also found that p66Shc silencing attenuated inflammatory response associated with the decline in mitochondrial oxidative stress. So p66Shc modulated CSE-induced airway epithelial cell injury and mitochondrial dysfunction. Nevertheless, it is still unclear what regulates p66Shc activity during airway epithelial cell injury induced by CSE.

PKC is constituted of serine/threonine phosphorylating enzymes whose activation is via a second messenger [36]. It is a superfamily of 11 isozymes classified into three classes based on their structural features and sensitivity to activators: (i) conventional or calcium-dependent cPKCs (*α*, *β*I, *β*II, and *γ*); (ii) novel or calcium-independent nPKCs (*δ*, *ε*, *η*, and *θ*); and (iii) atypical aPKCs (*ζ*, *ι*, and *λ*) [36]. Five isozymes of PKC (*α*, *β*, *δ*, *θ*, and *ζ*) are involved in the pathogenesis of pulmonary diseases by the regulation of several cellular responses including permeability, contraction, migration, hypertrophy, proliferation, apoptosis, and secretion [37]. It has been demonstrated that PKCs may play important roles in the development of COPD through the regulation of airway inflammation, mucus hypersecretion, and epithelial barrier [37]. For example, cigarette smoke phosphorylates c-Scr through a PKC-*α*-dependent mechanism to activate epidermal growth factor receptor and thereby induce mitogen-activated protein kinase response in human small airway epithelial cells [38]. The PKC*α*/*β* signaling pathway is involved in the regulation of surfactant protein A and D expression and epithelial barrier disruption in human airway epithelial cells stimulated with nicotine and *Pseudomonas aeruginosa* elastase, respectively [39, 40]. Some studies have shown that PKC*δ* is involved in human neutrophil elastase-induced mucin hypersecretion from human bronchial epithelial cells [41] and toll-like receptor 2 regulates barrier function of human bronchial epithelial monolayers through a PKC*ζ*-dependent mechanism [42]. However, the roles of PKCs in the mitochondrial dysfunction of airway epithelial cells are still unknown.

Previous studies have demonstrated that PKC*β* promotes the translocation of p66Shc into the mitochondria of various cells under oxidative stress [22, 32, 43]. In addition, phosphorylated PKC*δ* can also activate p66Shc in COS-7 and proximal tubular epithelial cells upon oxidative stress stimulation [23, 33]. Therefore, we speculated that PKC*β* and PKC*δ* might also regulate p66Shc activity and mitochondrial function when airway epithelial cells were exposed to CSE. In order to confirm our speculation, further experiments have been done. We have found that 7.5% CSE significantly increased the total and phosphorylated protein expression of PKC*β* and PKC*δ*, associated with mitochondrial dysfunction and cell injury. These results supported the notion that PKC*β* and PKC*δ* are involved in CSE-induced airway epithelial mitochondrial damage. Our present study has also revealed that inhibition of PKC*β* and PKC*δ* significantly decreased p66Shc activation and its mitochondrial translocation and reduced cytochrome c release from the mitochondria in airway epithelial cells stimulated with CSE, suggesting that PKC*β* and PKC*δ* are also the upstream regulatory molecules of p66Shc in airway epithelial cells. Associated with the decreased activation of p66Shc, inhibition of PKC*β* and PKC*δ* also successfully attenuated mitochondrial dysfunction, cell injury, and inflammation response in airway epithelial cells exposed to CSE.

However, there are some potential limitations in our present research. First, the mitochondria were isolated from airway epithelial cells by differential centrifugation, and they can be further purified by percoll gradient ultracentrifugation. Second, the data for the effects of p66Shc silencing or PKC inhibitors alone are absent, and these experiments will be done in our future studies. Lastly, particulate matters from cigarette smoke can be deposited in the lung, increase oxidative stress as well as inflammatory response, and aggravate the injury of airway epithelial cells. However, the CSE used in this study was filtered and particulate matters in cigarette smoke were mostly removed. Whole smoke exposure system will be used in the future studies, which enables cultured cells to be directly exposed to native and unmodified cigarette smoke at the air-liquid interface and ensures the exposure similar to physiological inhalation.

In conclusion, our present study has shown that the PKC*β*/*δ*-p66Shc signaling pathway modulated airway epithelial cell oxidative damage and mitochondrial dysfunction induced by CSE. These data suggest that a novel PKC*β*/*δ*-p66Shc signaling pathway may be involved in the pathogenesis of COPD and other oxidative stress-associated pulmonary diseases and provide a potential therapeutic target for these diseases.
